# Identification of Key Biomarkers and Immune Infiltration in Sciatic Nerve of Diabetic Neuropathy BKS-db/db Mice by Bioinformatics Analysis

**DOI:** 10.3389/fphar.2021.682005

**Published:** 2021-05-26

**Authors:** Yixuan Lin, Fanjing Wang, Lianzhi Cheng, Zhaohui Fang, Guoming Shen

**Affiliations:** ^1^ Graduate School of Anhui University of Chinese Medicine, Hefei, China; ^2^ Department of Endocrinology, The First Affiliated Hospital of Anhui University of Traditional Chinese Medicine, Hefei, China; ^3^ Anhui Academic of Traditional Chinese Medicine Diabetes Research Institute, Hefei, China

**Keywords:** bioinformatics analysis, immune infiltration, diabetic neuropathy, key biomarkers, sciatic nerve

## Abstract

Diabetic neuropathy (DN) is one of the chronic complications of diabetes which can cause severe harm to patients. In order to determine the key genes and pathways related to the pathogenesis of DN, we downloaded the microarray data set GSE27382 from Gene Expression Omnibus (GEO) and adopted bioinformatics methods for comprehensive analysis, including functional enrichment, construction of PPI networks, central genes screening, TFs-target interaction analysis, and evaluation of immune infiltration characteristics. Finally, we examined quantitative real- time PCR (qPCR) to validate the expression of hub genes. A total of 318 differentially expressed genes (DEGs) were identified, among which 125 upregulated DEGs were enriched in the mitotic nuclear division, extracellular region, immunoglobulin receptor binding, and p53 signaling pathway, while 193 downregulated DEGs were enriched in ion transport, membrane, synapse, sodium channel activity, and retrograde endocannabinoid signaling. GSEA plots showed that condensed nuclear chromosome kinetochore were the most significant enriched gene set positively correlated with the DN group. Importantly, we identified five central genes (Birc5, Bub1, Cdk1, Ccnb2, and Ccnb1), and KEGG pathway analysis showed that the five hub genes were focused on progesterone-mediated oocyte maturation, cell cycle, and p53 signaling pathway. The proportion of immune cells from DN tissue and normal group showed significant individual differences. In DN samples, T cells CD4 memory resting and dendritic cells resting accounted for a higher proportion, and macrophage M2 accounted for a lower proportion. In addition, all five central genes showed consistent correlation with immune cell infiltration levels. qPCR showed the same expression trend of five central genes as in our analysis. Our research identified key genes related to differential genes and immune infiltration related to the pathogenesis of DN and provided new diagnostic and potential therapeutic targets for DN.

## Introduction

Diabetic neuropathy (DN) is one of the most common and serious chronic complications of diabetic mellitus (DM) (Barrett et al., 2017; Iqbal et al., 2018), which is characterized by pain, paresthesia, and sensory loss (Pinzur, 2011). It has been stated that DN impacts approximately 60–70% of diabetics (Tesfaye et al., 2010; Callaghan et al., 2012), and if it cannot be treated well, it would multiply the disability and mortality of diabetics (Davies et al., 2006; Smith et al., 2012). However, in addition to controlling blood glucose levels, no effective treatment have been found to prevent, slow, or reverse the progression of DN (Charnogursky et al., 2014). Therefore, identifying molecular biomarkers and exploring the underlying mechanisms of DN pathogenesis is vital for early diagnosis, prognosis, and personalized treatment of DN.

The principal pathological process of DN includes axonal degeneration and segmental demyelination (Vinik et al., 2000). Studies have identified persistently impaired insulin function and hyperglycemia to cause a series of downstream abnormalities that eventually lead to axon loss (Malik et al., 2001; Sima and Zhang, 2014). In addition, several biological pathways including oxidative stress, inflammation, apoptosis, and autophagy are also involved in the development of diabetic neuropathy (Fernyhough and McGavock, 2014; Roman-Pintos et al., 2016; Chung et al., 2018). However, the process is still controversial due to its complexity.

During the last decades, bioinformatics analysis has been extensively applied to analyze microarray data to identify differentially expressed genes (DEGs) and perform various analyses (Normand and Yanai, 2013; Servant et al., 2014). Integrating and reanalyzing these genomic data offer possibilities for identifying certain disease-related biomarkers. At present, a great deal of research has been conducted on the regulatory genes of DN (Guo et al., 2019; Zhou and Zhang, 2019; Jian and Yang, 2020). Comprehensive analysis of the expression profile data of these genes on the microarray platform will help us understand the pathogenesis more deeply and accurately. Selecting different high-throughput sequencing platforms and samples will cause differences in DEG identification results. In this study, we downloaded GSE27382 (Pande et al., 2011) gene chip from the Gene Expression Omnibus (GEO) database (Edgar et al., 2002) and screened DEGs of the sciatic nerve in DN model mouse and normal mouse to determine key biomarkers. Gene Ontology (GO) functional annotation analysis (Ashburner et al., 2000) and Kyoto Encyclopedia of Genes and Genomes (KEGG) pathway enrichment analysis (Wixon and Kell, 2000) were performed for the screened DEGs. The protein–protein interaction (PPI) network of DEGs was established by STRING (Szklarczyk et al., 2015) and visualized by Cytoscape software (Saito et al., 2012). We also constructed transcription factor (TF) regulation network (Han et al., 2018) and selected hub genes. Furthermore, we used the CIBERSORT algorithm (Chen et al., 2018) to predict the proportion of immune infiltration in sciatic nerve samples and analyzed the correlation between the hub gene and immune infiltration. At last, the mRNA expression levels of these five genes were verified by *in vitro* experimental analysis. Hopefully, our exploration is expected to provide novel clues for the diagnosis and potential therapeutic targets of DN.

## Materials and Methods

### Microarray Data Information

Gene Expression Omnibus (GEO, http://www.ncbi.nlm.nih.gov/geo) is a public genomics data repository of massive high throughput gene expression data (Edgar et al., 2002). The GSE27382 dataset provided by Manjusha Pande et al. (Pande et al., 2011) included 6 DN model mouse sciatic nerve and 7 normal mouse sciatic nerve. They assessed the neuropathy of BKS db/db mouse (a genetic mouse with spontaneous type 2 diabetes) and prepared a model of DN. Heterozygous (db/+) mice do not develop diabetes and were used as no diabetic controls in experimental DN. Microarray technology was performed to compare RNA expression of mouse sciatic nerve. Affymetrix GeneChip Mouse Genome 430 2.0 Array (CDF: Mouse4302_Mm_ENTREZG.cdf version 12.0.0) was used as the platform.

### Data Pre-Processing and DEG Identification

We used R software (version 3.6.3; https:// www.r‐project.org/) and Bioconductor packages (http://www.bioconductor.org/) for data correction and analysis. The probe name in the MINiML file was translated into the gene symbol by the R package and saved as a TXT file. The standardized data matrix was analyzed using the limma R software package. | logFC (fold change) | > 2 and adjusted *p*-value < 0.05 were considered statistically significant, aiming at reducing the false positive rate. Then, we used TBtools software (Chen et al., 2020) (http://cj-chen.github.io/tbtools/) to visualize the raw data in TXT format and drawn cluster heat map and volcano plot.

### GO and KEGG Pathway Enrichment Analyses

GO annotates and classifies gene sets through biological pathways (BP), cellular components (CC), and molecular function (MF), while KEGG hints biological pathways related to DEGs (Ashburner et al., 2000; Wixon and Kell, 2000). Gene set enrichment analysis (GSEA) is a computational method that determines whether there is a significant difference between two groups for a defined set of genes. The DAVID online database provides a comprehensive set of functional annotation tools for annotation, visualization, and integrated discovery to understand the biological meaning of genes and proteins. GO and KEGG analysis in the present study was performed using (DAVID, http://david.ncifcrf.gov) (version 6.8) and GSEA software (version 3.0). The cut-off criterion was set as *p* < 0.05 and gene counts ≥ 5 (Huang et al., 2007).

### PPI Network Construction

The PPI network was established by a Search Tool for the Retrieval of Interacting Genes (STRING; http://string-db.org) (version 10.0) online database (Szklarczyk et al., 2015), and combined score > 0.4 was considered a significant interaction. Meanwhile, we applied Cytoscape software (https://cytoscape.org/) (version 3.6.1) to visualize the PPI network (Saito et al., 2012).

### Module Analysis and CytoHubba Analysis

Molecular Complexity Detection (MCODE), a plugin in Cytoscape, identifies the most important modules in a PPI network based on nodes and scores (Anitha et al., 2016). MCODE scores > 5, degree cutoff = 2, node score cutoff = 0.2, K-core = 2, and Max depth = 100 were set as filtering parameters. Another plugin, CytoHubba (Chin et al., 2014), was used to screen out key subnets. We used the maximal clique centrality (MCC) algorithm to rank the nodes for network centrality and select the top 20 as candidate genes.

### TF Regulation Prediction

TRRUSTv2 (http://www.grnpedia.org/trrust/) is a manually curated database of human and mouse transcriptional regulatory interactions. This database used a continuously improved sentence-based text mining algorithm, coupled with careful manual proofreading after mining, to ensure that the interaction between the TFs-targets in the database was experimentally verified (Han et al., 2018). Based on this online database, we found regulatory factors of DEGs and comprehensively analyzed the interaction between TF and its target genes. Then, we screened the target genes and TFs linked to target genes to build a co-expression network.

### Screening for Hub Genes

The MCC algorithm of CytoHubba, MCODE scoring method, and key TF target genes were used to comprehensively screen the important hub genes among DEGs. We employed the web tool Venn diagram (http://bioinfogp.cnb.csic.es/tools/venny/) to obtain the common hub genes in three different experiments. Subsequently, the biological process analysis of hub genes was performed using Biological Networks Gene Ontology tool (BiNGO) (version 3.0.3) (Maere et al., 2005) plugin of Cytoscape, and we reanalyzed hub genes *via* KEGG pathway enrichment.

### Determination of Immune Cell Landscapes

We use the bioinformatics algorithm CIBERSORT to calculate the proportion of 22 immune cells based on the gene expression profile. The 22 infiltrating immune cells include B cells, T cells, natural killer cells, macrophages, dendritic cells, and myeloid subsets (Edgar et al., 2002; Newman et al., 2015; Chen et al., 2018). The CIBERSORT analysis tool uses Monte Carlo sampling to obtain the P-value of the deconvolution of each sample to determine the confidence. At *p* < 0.05, the inferred CIBERSORT immune cell population score was accurate. We uploaded the standardized gene expression data set to the CIBERSORT Web site (http://cibersort.stanford.edu/), with the permutation parameter to 1,000, screened out samples with *p* < 0.05, calculated the percentage of each immune cell in the sample, analyzed the immune infiltration level of each immune cell between the two groups, and performed principal component analysis (PCA). The immune cell type fractions summed up to one. Finally, we used the Pearson method to analyze the relationship between immune cells and core gene expression.

### qRT-PCR Assay of the Hub Genes

The RSC96 cell line was purchased from the Institute of Biochemistry and Cell Biology, CAS (Shanghai, China). The cells were cultured in Dulbecco’s modified Eagle’s medium (DMEM; Gibco, Grand Island, NY, United States) containing 10% FBS (Gibco), 100 mg/ml streptomycin and 100 U/ml penicillin (Biyuntian, China) at 37°C in a humidified 5% CO2 incubator (Thermo, Waltham, MA, United States). After the cells were fused to 70–80%, they were digested with 0.25% trypsin for subsequent experiments. The cells were divided into two groups: control group and model group. The glucose concentration in DMEM is 5.6 mM, which is considered normal glucose (Con), and 50 mM is considered high glucose (HG). The normal group was cultured at a concentration of 5.6 mM glucose. The glycotoxicity model group was first cultured under 5.6 mM glucose for 24 h, and then incubated with 50 mM glucose for 48 h. The equivalent concentration of mannitol was used as an osmotic control.

We extracted total RNA from two groups of cells using TRIzol reagent (Invitrogen, Thermo Fisher Scientific, Inc.). RNA samples from total RNA were reverse transcribed to cDNA, and qRT-PCR was carried out using the 7500 Real-Time PCR instrument (Applied Biosystems, United States). The expression levels of the selected genes were normalized against β-actin. The PCR primers used in this study are displayed in Table 1. Student’s *t* test was used for the statistical analysis, and *p* < 0.05 indicated a significant difference.

**TABLE 1:** Primers used in qRT-PCR experiments.

Gene	Forward primer (5′-3′)	Reverse primer (5′-3′)
Birc5	TGC​CCT​ACC​GAG​AAT​GAG​C	TTC​CAC​CTG​CTT​CTT​GAC​TGT
Bub1	GAA​AAC​TCA​GCT​TGC​GTT​CC	AGG​CTT​GGG​TGC​CAT​AGA​T
Cdk1	CAG​GAC​TCC​AGG​CTG​TAT​CTC	TCG​GTA​TTC​CAA​ACG​CTC​T
Ccnb1	CAG​AGG​TGG​AAC​TGG​ATG​AGC	CAC​ATC​GGA​GAA​AGC​CTG​ACA
Ccnb2	CAT​TCC​AAG​TTT​AGG​CTT​CTG​C	GAT​TTG​GGA​ACT​GGT​GTA​AGC​A
β-actin	CGT​TGA​CAT​CCG​TAA​AGA​C	TAGGAGCCAGGGCAGTA

## Results

### Identification of DEGs

**FIGURE 1:**
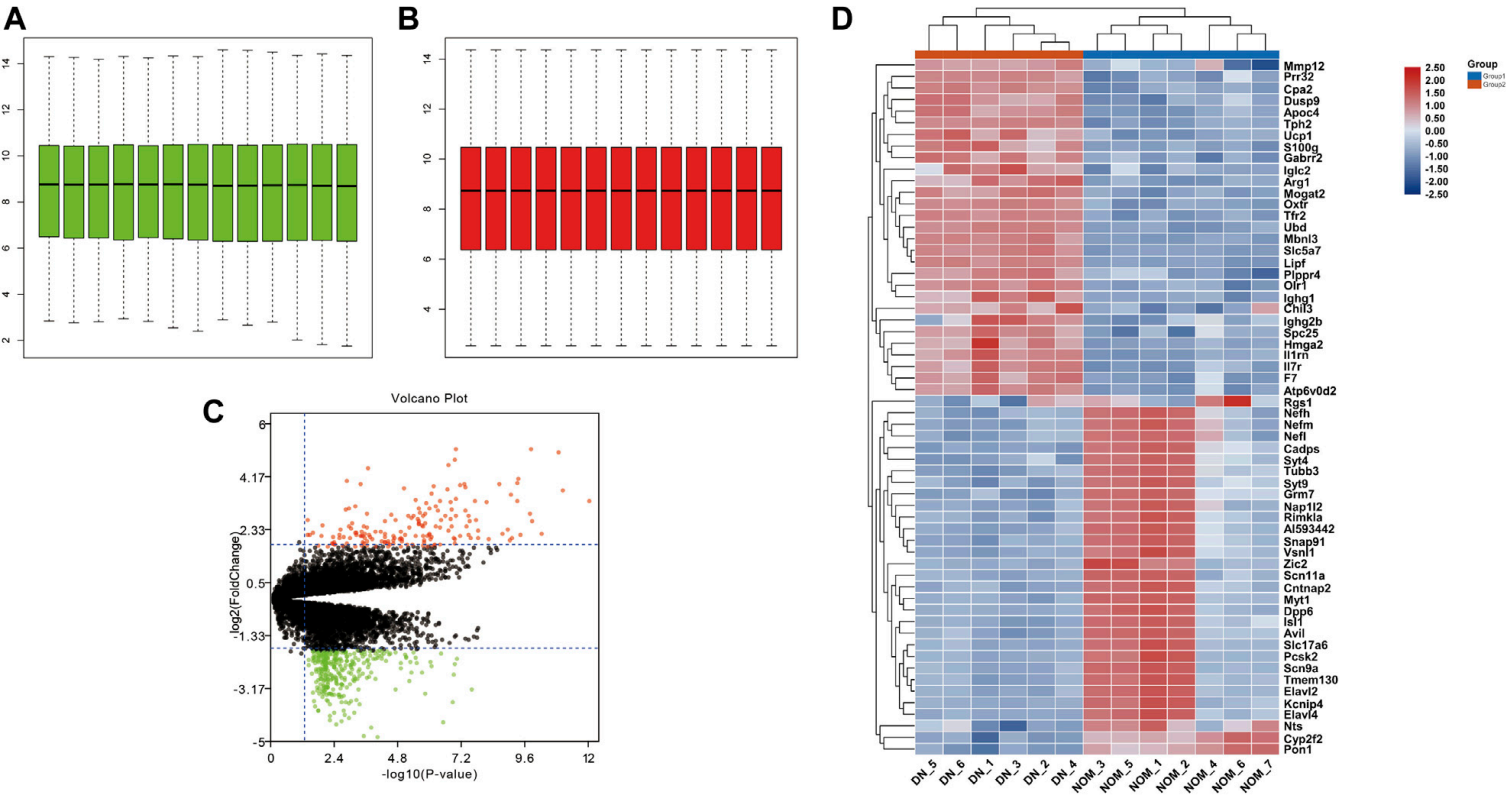
Standardization of GSE27382. **(A,B)** The green bar represents the data before normalization, and the red bar represents the data after normalization. **(C)** Volcano plots of DEGs in GSE27382. Red plots represent the upregulated genes with logFC > 2 and adjusted *p*-value < 0.05. Green plots represent the downregulated genes with logFC < −2 and adjusted *p*-value < 0.05. Black plots represent the remaining genes with no significant difference. **(D)** Heat maps of DEGs display of the most significant top 50 upregulated and downregulated genes.

After normalizing the microarray results from GSE27382 (Figures 1A,B), we identified 318 DEGs including 125 upregulated genes (logFC > 0) and 193 downregulated genes (logFC < 0) after the comparison between 6 DN samples and 7 normal sciatic nerve samples. The identified DEGs were showed by volcano plot (Figure 1C), the top 30 upregulated and downregulated genes of the two expression groups were displayed in a heat map (Figure 1D).

### GO and KEGG Pathway Enrichment Analyses

We performed functional analysis of DEGs by the DAVID online software (Table 2). In the GO enrichment analysis, the upregulated DEGs in BP were mainly enriched in mitotic nuclear division, cell division, inflammatory response, cell cycle, chromosome segregation, and innate immune response, while the downregulated DEGs in BP were enriched in ion transport, transport, sodium ion transport, synaptic vesicle exocytosis, retinal ganglion cell axon guidance, and learning. CC analysis showed that the upregulated DEGs were significantly enriched in the extracellular region, extracellular space, condensed chromosome kinetochore, kinetochore, chromosome, centromeric region, and external side of plasma membrane, while the downregulated DEGs were enriched in the membrane, synapse, axon, neuronal cell body, perikaryon, and synaptic vesicle. As for MF, the upregulated DEGs were mainly focused on immunoglobulin receptor binding, serine-type endopeptidase activity, peptidase activity, calcium ion binding, serine-type peptidase activity, and peptide hormone binding, and downregulated DEGs were mainly focused on sodium channel activity, voltage-gated sodium channel activity, voltage-gated ion channel activity, clathrin binding, potassium channel regulator activity, and calcium ion binding. Additionally, KEGG pathway analysis results are displayed in Table 3. We discovered that upregulated DEGs were significantly enriched in p53 signaling pathway, complement and coagulation cascades, progesterone-mediated oocyte maturation, oocyte meiosis, neuroactive ligand-receptor interaction, and cell cycle, whereas downregulated DEGs were significantly enriched in retrograde endocannabinoid signaling, dopaminergic synapse, nicotine addiction, amyotrophic lateral sclerosis (ALS), synaptic vesicle cycle, and neuroactive ligand-receptor interaction.

**TABLE 2:** Gene ontology enrichment analysis of differentially expressed genes.

Expression	Category	Term	Count	*p*-value
Downregulated	GOTERM_BP_DIRECT	GO:0006811∼ion transport	22	1.30E-07
	GOTERM_BP_DIRECT	GO:0006810∼transport	40	5.91E-07
	GOTERM_BP_DIRECT	GO:0006814∼sodium ion transport	10	2.49E-06
	GOTERM_BP_DIRECT	GO:0016079∼synaptic vesicle exocytosis	5	2.56E-05
	GOTERM_BP_DIRECT	GO:0031290∼retinal ganglion cell axon guidance	5	3.17E-05
	GOTERM_BP_DIRECT	GO:0007612∼learning	7	3.48E-05
	GOTERM_CC_DIRECT	GO:0016020∼membrane	109	3.10E-11
	GOTERM_CC_DIRECT	GO:0045202∼synapse	24	2.84E-10
	GOTERM_CC_DIRECT	GO:0030424∼axon	20	1.58E-09
	GOTERM_CC_DIRECT	GO:0043025∼neuronal cell body	23	4.55E-09
	GOTERM_CC_DIRECT	GO:0043204∼perikaryon	12	1.46E-07
	GOTERM_CC_DIRECT	GO:0008021∼synaptic vesicle	11	2.61E-07
	GOTERM_MF_DIRECT	GO:0005272∼sodium channel activity	6	7.90E-06
	GOTERM_MF_DIRECT	GO:0005248∼voltage-gated sodium channel activity	5	3.17E-05
	GOTERM_MF_DIRECT	GO:0005244∼voltage-gated ion channel activity	9	3.76E-05
	GOTERM_MF_DIRECT	GO:0030276∼clathrin binding	6	6.73E-05
	GOTERM_MF_DIRECT	GO:0015459∼potassium channel regulator activity	5	2.74E-04
	GOTERM_MF_DIRECT	GO:0005509∼calcium ion binding	18	2.98E-04
	GOTERM_BP_DIRECT	GO:0006811∼ion transport	22	1.30E-07
	GOTERM_BP_DIRECT	GO:0006810∼transport	40	5.91E-07
	GOTERM_BP_DIRECT	GO:0006814∼sodium ion transport	10	2.49E-06
	GOTERM_BP_DIRECT	GO:0016079∼synaptic vesicle exocytosis	5	2.56E-05
Upregulated	GOTERM_BP_DIRECT	GO:0007067∼mitotic nuclear division	13	1.82E-07
	GOTERM_BP_DIRECT	GO:0051301∼cell division	13	4.27E-06
	GOTERM_BP_DIRECT	GO:0006954∼inflammatory response	11	6.44E-05
	GOTERM_BP_DIRECT	GO:0007049∼cell cycle	14	1.31E-04
	GOTERM_BP_DIRECT	GO:0007059∼chromosome segregation	5	2.42E-03
	GOTERM_BP_DIRECT	GO:0045087∼innate immune response	9	3.84E-03
	GOTERM_CC_DIRECT	GO:0005576∼extracellular region	30	3.56E-07
	GOTERM_CC_DIRECT	GO:0005615∼extracellular space	26	2.53E-06
	GOTERM_CC_DIRECT	GO:0000777∼condensed chromosome kinetochore	5	1.64E-03
	GOTERM_CC_DIRECT	GO:0000776∼kinetochore	5	5.93E-03
	GOTERM_CC_DIRECT	GO:0000775∼chromosome, centromeric region	5	1.04E-02
	GOTERM_CC_DIRECT	GO:0009897∼external side of plasma membrane	7	1.22E-02
	GOTERM_MF_DIRECT	GO:0034987∼immunoglobulin receptor binding	4	8.90E-04
	GOTERM_MF_DIRECT	GO:0004252∼serine-type endopeptidase activity	7	1.96E-03
	GOTERM_MF_DIRECT	GO:0008233∼peptidase activity	10	4.46E-03
	GOTERM_MF_DIRECT	GO:0005509∼calcium ion binding	11	1.07E-02
	GOTERM_MF_DIRECT	GO:0008236∼serine-type peptidase activity	5	1.80E-02

**TABLE 3:** Kyoto Encyclopedia of Genes and Genomes (KEGG) pathway analysis of differentially expressed genes.

Expression	Category	Term	Count	*p*-Value	Genes
Downregulated	KEGG_PATHWAY	mmu04723:Retrograde endocannabinoid signaling	6	8.68E-04	SLC17A7, GABRG1, GABRG2, SLC17A6, GNG3, MAPK10
	KEGG_PATHWAY	mmu04728:Dopaminergic synapse	6	0.002792254	GNAL, SCN1A, GNG3, MAPK10, PPP2R2B, PPP2R2C
	KEGG_PATHWAY	mmu05033:Nicotine addiction	4	0.002985375	SLC17A7, GABRG1, GABRG2, SLC17A6
	KEGG_PATHWAY	mmu05014:Amyotrophic lateral sclerosis (ALS)	4	0.005947408	PRPH, NEFH, NEFL, NEFM
	KEGG_PATHWAY	mmu04721:Synaptic vesicle cycle	4	0.010210301	SLC17A7, SLC17A6, CPLX1, ATP6V1G2
	KEGG_PATHWAY	mmu04080:Neuroactive ligand-receptor interaction	7	0.016857166	GABRG1, F2RL2, ADRB3, GABRG2, AGTR1B, GRM7, NPY2R
Upregulated	KEGG_PATHWAY	mmu04115:p53 signaling pathway	4	0.00976751	CCNB1, CDK1, CCNB2, SERPINE1
	KEGG_PATHWAY	mmu04610:Complement and coagulation cascades	4	0.013749751	C6, SERPINE1, F7, CFI
	KEGG_PATHWAY	mmu04914:Progesterone-mediated oocyte maturation	4	0.019706172	CCNB1, CDK1, CCNB2, BUB1
	KEGG_PATHWAY	mmu04114:Oocyte meiosis	4	0.037781539	CCNB1, CDK1, CCNB2, BUB1
	KEGG_PATHWAY	mmu04080:Neuroactive ligand-receptor interaction	6	0.039529327	GABRR2, AGTR2, MC2R, OXTR, TSHR, CHRNA2
	KEGG_PATHWAY	mmu04110:Cell cycle	4	0.048667279	CCNB1, CDK1, CCNB2, BUB1

**FIGURE 2:**
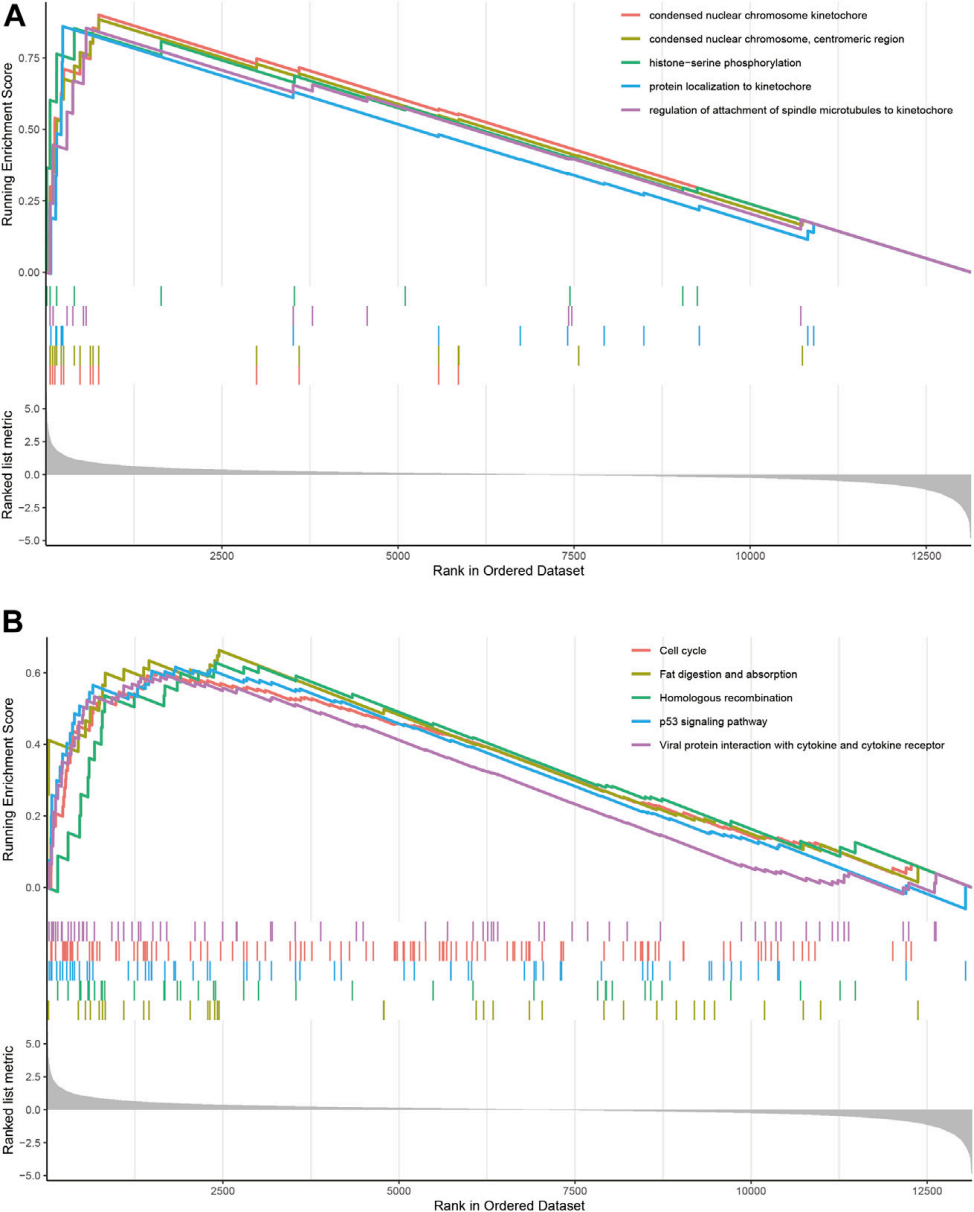
GSEA identified GO and KEGG pathways enriched in the DN group and normal controls. **(A)** GO analysis of the top five significantly enriched gene set in DN group. **(B)** KEGG analysis of the top five significantly enriched pathways in DN group.

GSEA identified 1,204 GO terms and 26 KEGG terms in DN group and normal controls. GO analysis indicated that the condensed nuclear chromosome kinetochore, condensed nuclear chromosome, centromeric region, protein localization to kinetochore, regulation of attachment of spindle microtubules to kinetochore, and histone–serine phosphorylation were the most significantly enriched biological processes in DN group (Figure 2A). Most significant enriched pathway positively correlated with the DN group included fat digestion and absorption, homologous recombination, p53 signaling pathway, cell cycle, and viral protein interaction with cytokine and cytokine receptor (Figure 2B).

### PPI Network Analysis

**FIGURE 3:**
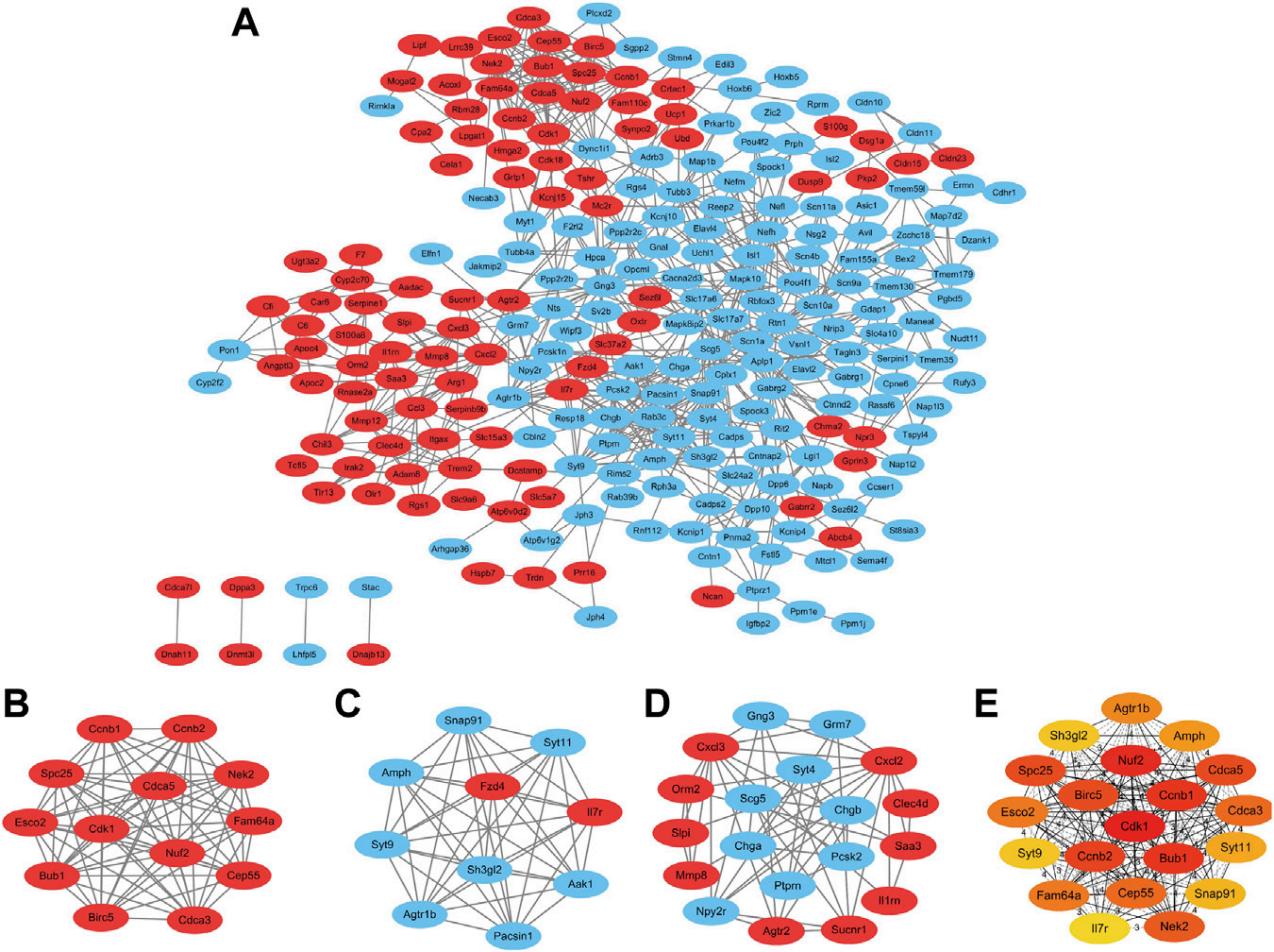
Protein–protein interaction network construction and analysis of differentially expressed genes. **(A)** Red nodes represent upregulated genes, and blue nodes represent downregulated genes. **(B–D)** Three significant modules with MCODE score > 5 and top 20 maximal clique centrality (MCC) protein nodes. Three modules extracted from the PPI network. Red nodes indicate upregulated DEGs and green nodes indicate downregulated DEGs. **(E)** Top 20 candidate genes with maximal clique centrality.

We constructed a protein interactions network by the STRING online platform and extracted the proteins with a combined score > 0.4 for visualization by Cytoscape. A total of 247 DEGs from 318 candidate DEGs were contained into the network which included 247 nodes and 746 edges (Figure 3A). Among them, the red nodes represent 98 upregulated genes, and the blue nodes represent 149 downregulated genes. The remaining 71 DEGs do not interact with any other DEGs, so they are not included in the PPI network.

### Module Analysis and CytoHubba Analysis

We analyzed the total nodes by the Cytoscape plugin MCODE and identified three significant modules with an MCODE score > 5 (Table 4). Module 1 contains 13 nodes and 77 edges (Figure 3B); Module 2 contains 10 nodes and 55 edges (Figure 3C); Module 3 contains 19 nodes and 54 edges (Figure 3D). The most significant Module A consisted of 13 nodes (Fam64a, Ccnb1, Cdk1, Bub1, Nuf2, Ccnb2, Spc25, Birc5, Cdca5, Nek2, Cdca3, Cep55, and Esco2). CytoHubba plugin was used to explore the important nodes and sub-networks in the network. The top 20 maximal clique centrality (MCC) protein nodes were selected as candidate genes. CytoHubba plugin was used to explore the pivotal nodes and sub-networks in the network. The top 20 maximal clique centrality (MCC) protein nodes were selected as candidate genes, including Agtr1b, Amph, Birc5, Bub1, Ccnb1, Ccnb2, Cdca3, Cdca5, Cdk1, Cep55, Esco2, Fam64a, Il7r, Nek2, Nuf2, Sh3gl2, Snap91, Spc25, Syt11, and Syt9 (Figure 3E).

**TABLE 4:** Three most significant modules from the PPI networks with MCODE score > 5.

Cluster	Score	Nodes	Edges	Node IDs
1	11	13	77	Fam64a, Ccnb1, Cdk1, Bub1, Nuf2, Ccnb2, Spc25, Birc5, Cdca5, Nek2, Cdca3, Cep55, and Esco2
2	10	10	45	Sh3gl2, Amph, Pacsin1, Snap91, Aak1, Syt11, Agtr1b, Syt9, Fzd4, and Il7r
3	6	19	54	Ptprn, Pcsk2, Scg5, Chgb, Chga, Cxcl2, Mmp8, Clec4d, Cxcl3, Slpi, Agtr2, Gng3, Npy2r, Orm2, Grm7, Sucnr1, Syt4, Il1rn, and Saa3

### TF Regulatory Network Analysis

**FIGURE 4:**
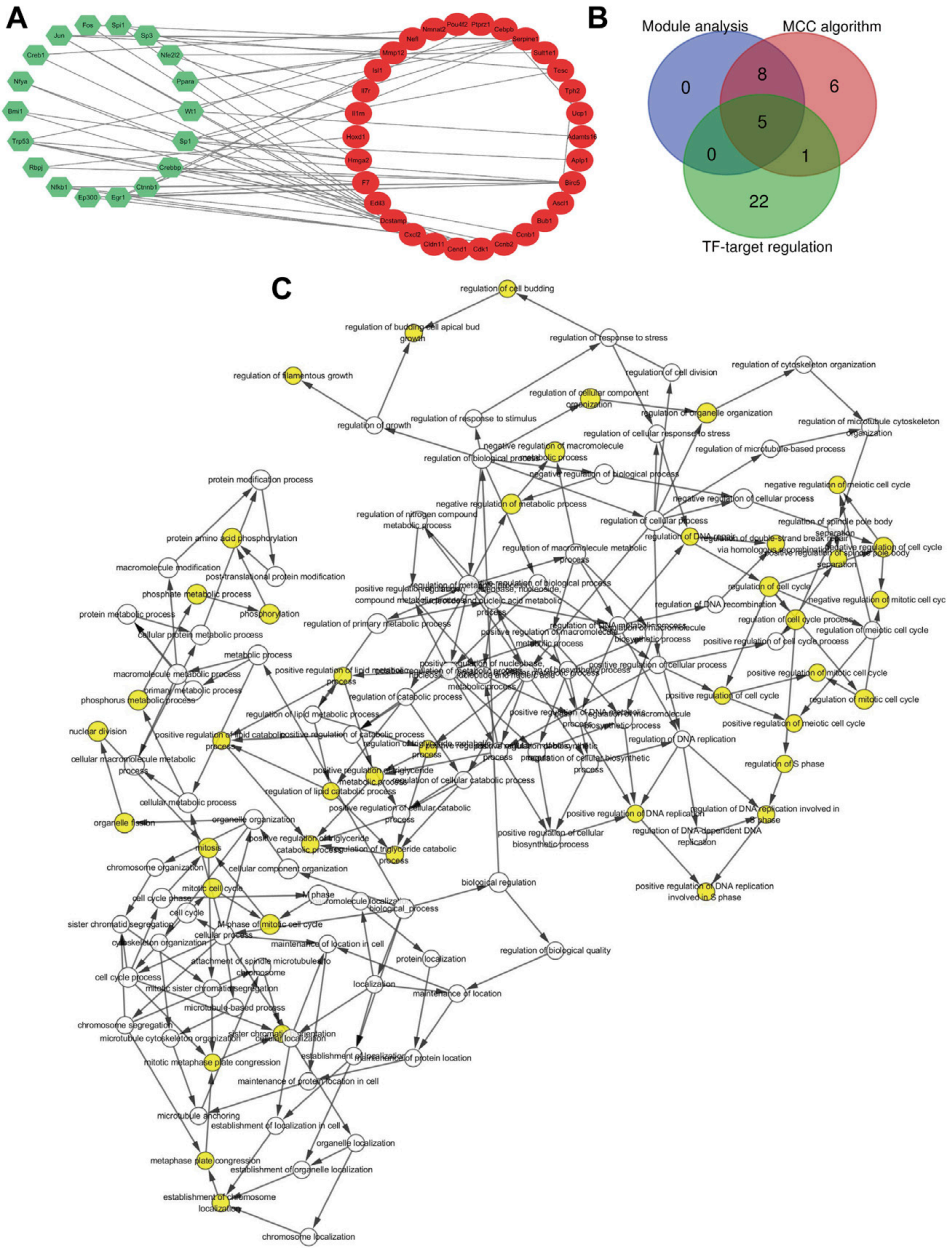
Gene transcription factor and hub genes identified. **(A)** Gene transcription factor (TF) regulatory network. Green hexagons stand for the transcription factor and red nodes stands for DEGs. **(B)** Hub genes identified by Venn diagram. The blue circle represents the 13 significant genes generated by module analysis, the red circle represents the top 20 candidate genes obtained by the MCC algorithm, and the green circle represents the 28 target genes predicted by TFs. **(C)** GO analysis of the hub gene constructed using BiNGO. The color depth of the node refers to the corrected *p*-value of the ontology. The size of the node refers to the number of genes involved in the ontology. *p* < 0.01 is statistically significant.

According to the TRRUSTv2 database, we calculated the TFs that regulate DEG and obtained TFs-target interaction information. A gene-TF regulatory network was constructed including 28 genes and 21 TFs, resulting in 55 recombination relationship pairs (Figure 4A). While Birc5 and Serpine1 were found to be regulated by 7 TFs; Dcstamp was regulated by 4 TFs, Cxcl2, and Hmga2 were regulated by 3 TFs; Ccnb2, Cdk1, Cldn11, F7, Mmp12, Nefl, Tesc, Ucp1 was regulated by 2 TFs.

### Hub Gene Selection and Analysis

In the end, the 13 significant genes generated by module analysis, the top 20 candidate genes obtained by the MCC algorithm, and the 28 target genes predicted by TFs were applied to the overlap of Venn diagrams (Figure 4B). We identified five common genes from three groups, including Birc5, Bub1, Cdk1, Ccnb2, and Ccnb1. Therefore, these hub genes may serve as promising biomarkers of DN. The GO analysis of five hub genes using BiNGO is shown in Figure 4C, the KEGG pathway results showed that the hub genes were significantly enriched in progesterone-mediated oocyte maturation, cell cycle, p53 signaling pathway, and oocyte meiosis (Table 5).

**TABLE 5:** Reanalysis hub genes via KEGG pathway enrichment analysis.

Category	Term	Count	*p*-Value	Genes
KEGG_PATHWAY	cfa04914:Progesterone-mediated oocyte maturation	4	7.92E-06	CCNB1, CDK1, CCNB2, BUB1
KEGG_PATHWAY	cfa04110:Cell cycle	4	2.33E-05	CCNB1, CDK1, CCNB2, BUB1
KEGG_PATHWAY	cfa04115:p53 signaling pathway	3	5.41E-04	CCNB1, CDK1, CCNB2
KEGG_PATHWAY	cfa04114:Oocyte meiosis	3	1.49E-03	CDK1, CCNB2, BUB1

### Determination of Immune Infiltration

**FIGURE 5:**
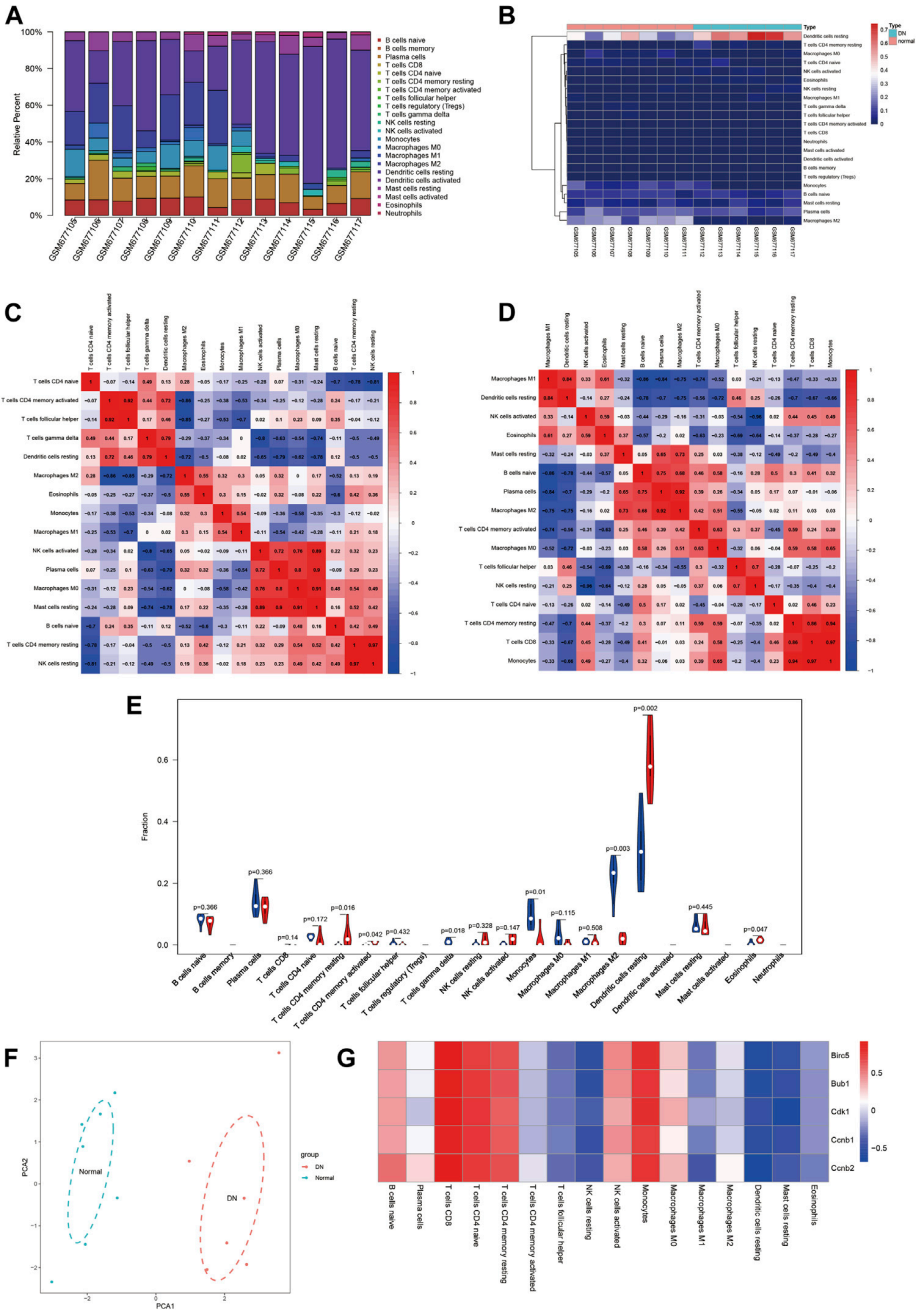
Landscape of immune infiltration between DN samples and normal samples. **(A)** The bar plot represents the relative percentage of 22 immune cells in each sample. **(B)** Cluster heat map based on 22 immune cell proportions of normal samples and DN samples. Red indicates a high percentage of immune cells and blue indicates a low percentage of immune cells. **(C,D)** Correlation matrix of the proportion of immune cells in the normal and DN groups. Red indicates positive correlation and blue indicates negative correlation. **(E)** The difference in immune infiltration between the normal and DN samples (blue for the normal group and red for the DN group, *p* value < 0.05 was considered statistically significant. **(F)** Principal component analysis of normal and DN samples. **(G)** Correlation index analysis of hub genes expression and immune infiltration level. Red indicates positive correlation and blue indicates negative correlation.

According to the CIBERSORT algorithm, 13 samples including normal samples (n = 7) and DN samples (n = 6) all meet the requirements of CIBERSORTP < 0.05. We used R software to visualize the profile of immune infiltration of 13 sciatic nerve tissues (Figure 5). Figures 5A,B show the relative proportions and stratified clustering of the 22 immune cells in each sample. Among them, dendritic cells resting account for most of all infiltrating cells, especially in DN tissues. Figures 5C,D show the correlation between the 22 immune infiltrating cells in normal and DN groups. In normal samples, T cells follicular helper and T cells CD4 memory activated, T cells CD4 memory resting and NK cells resting, B cells naive and plasma cells, and macrophage M0 showed strong correlation. In DN samples, macrophages M2 and plasma cells, monocytes and T cells CD4 memory resting, and T cells CD8 all showed significant positive correlations. Figure 5E further analyzes the differences in immune cells between normal and DN samples. Obviously, the P values of T cells CD4 memory resting, T cells CD4 memory activated, T cells gamma delta, monocytes, macrophages M2, dendritic cells resting, and eosinophils are 0.016, 0.042, 0.018, 0.01, 0.003, 0.002, and 0.047, respectively. There are significant differences in the proportions of these seven immune cells. Compared with normal tissues, DN tissues contain a higher proportion of T cells CD4 memory resting and dendritic cells resting and a lower proportion of macrophage M2. Figure 5F used principal component analysis (PCA) to reveal the significant cluster bias clustering and individual differences of the 22 immune cells in the two groups of samples. The results showed that the expression level of the normal group and the DN group were clearly differentiated. In summary, these findings indicated that the difference in the expression of immune infiltration between the DN group and the normal group has important clinical significance.

### Correlation Analysis of Hub Gene and Immune Infiltration

We investigated whether the expression of central genes in DN is related to immune infiltration. The results showed that all five central genes showed a consistent correlation with immune cell infiltration levels (Figure 5G). The expression of Hub gene was positively correlated with the infiltration level of T cells CD8, T cells CD4 naive, T cells CD4 memory resting, and monocytes, and had a large negative correlation with the infiltration level of dendritic cells resting. These results indicated that immune cell infiltration has an effect on DN progression, and Birc5, Bub1, Cdk1, Ccnb2, and Ccnb1 play an important role in DN immune infiltration.

### qRT-PCR Assay of the Hub Genes

**FIGURE 6:**
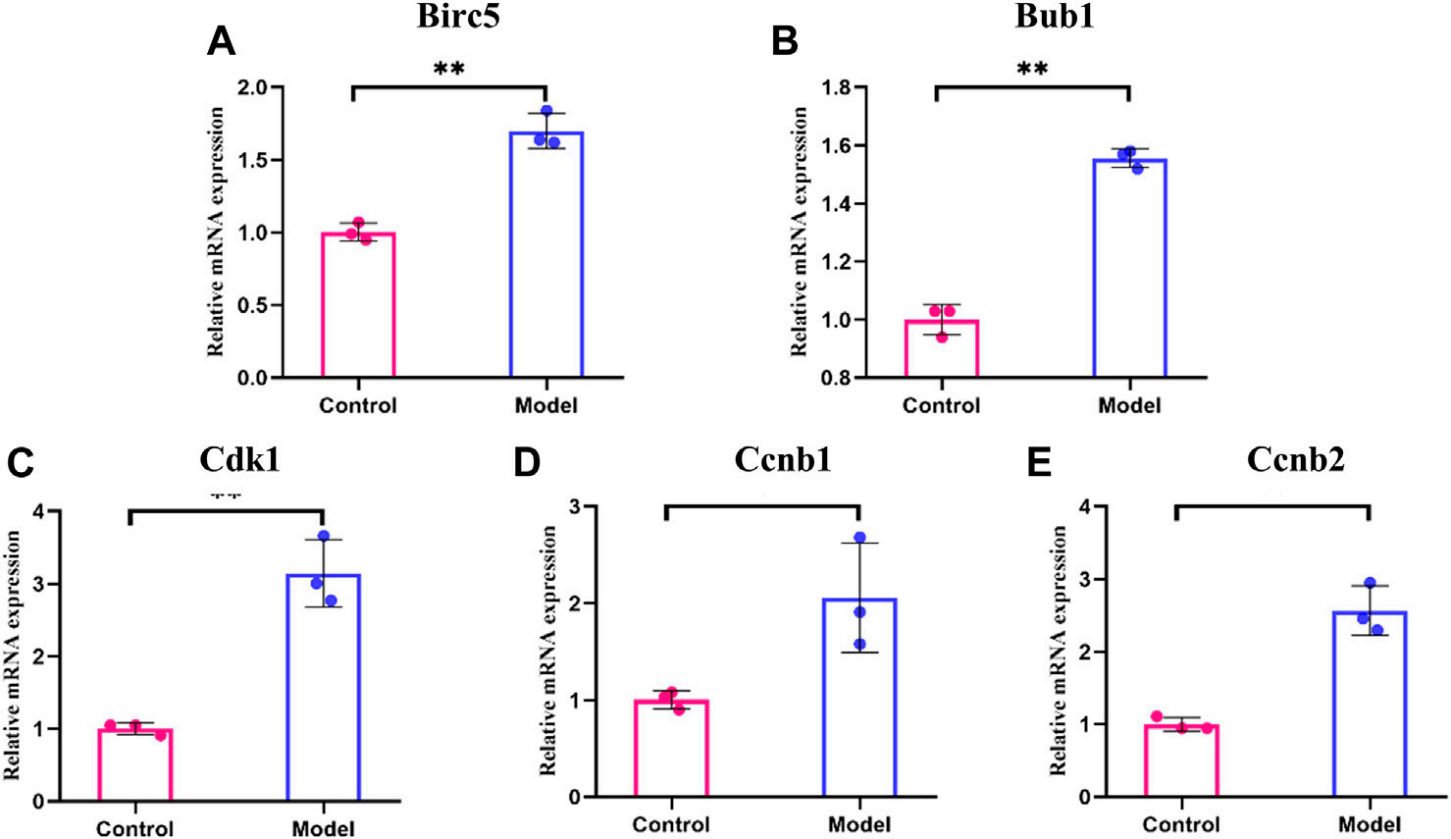
The mRNA levels for hub genes in glycotoxicity model and control group. **(A–E)** The transcript levels of Birc5, Bub1, Cdk1, Ccnb1 and Ccnb2 were significantly higher in the glycotoxicity model group compared to the control group. All experiments were repeated three times (n = 3). Mean ± SD., *p < 0.05, **p < 0.01.

We detected the expression of these five genes at the mRNA level by qRT-PCR analysis. The results showed that the transcript levels of Birc5, Bub1, Cdk1, Ccnb1, and Ccnb2 were significantly higher in the glycotoxicity model group compared to the control group (Figure 6, *p* < 0.05), which is consistent with our above bioinformatics analysis results.

## Discussion

Diabetes is regarded as a global threat to human health. According to the data from the International Diabetes Federation (IDF), by 2045, there will be approximately 700 million patients with diabetes (Saeedi et al., 2019). DN is the most insidious and long-term complication of diabetes, in addition to neuropathic symptoms, the secondary complications may lead to serious further morbidity including ulceration, fractures, amputations, and even death (Sung et al., 2012). Because of its complex pathophysiological pathway, there are currently no specific and efficient diagnostic methodologies and treatment strategies. Hence, it is greatly needed to excavate the DN‐related markers and therapeutic targets. RNA sequencing has been widely used in the research of endocrine and metabolic diseases (Wang et al., 2018; Backman et al., 2019). Studies have been conducted to analyze the protein markers of diabetic nephropathy (Caramori et al., 2015), diabetic retinopathy (Cui et al., 2018), diabetic encephalopathy (Song et al., 2017), and other complications using microarrays in diabetic patients or animal models. These explorations have achieved remarkable achievements and clinical benefits.

In this study, in order to investigate the change of expression profile in DN and reveal its biological process, the 13 samples from GSE27382 were selected to study the gene expression of the sciatic nerve. A total of 318 DEGs were identified between DN samples and normal sciatic nerve samples including 125 upregulated genes and 193 downregulated genes. Then, GO and KEGG pathways enrichment analyses of DEGs were performed. We constructed the PPI network and applied module and maximal clique centrality analysis. Furthermore, we predicted the TF-mediated transcriptional regulatory network and comprehensively filtered out five hub genes including Birc5, Bub1, Cdk1, Ccnb1, and Ccnb2. The qRT-PCR verification revealed that the relative transcription levels of these genes showed the same expression trends as in our bioinformatics analysis. Finally, using the CIBERSORT algorithm, we found that the proportion of immune infiltration in the DN and normal groups was different. In DN samples, T cells CD4 memory resting and dendritic cells resting accounted for a higher proportion, and macrophage M2 accounted for a lower proportion. The difference in DN immune spectrum may become a new target for immunotherapy.

Birc5, baculoviral IAP repeat-containing 5, is a member of the inhibitor of apoptosis (IAP) gene family, which encode negative regulatory proteins that prevent apoptotic cell death (Blanc-Brude et al., 2002). It is reported that BIRC5 can be involved in cell-cycle regulation and apoptosis by inhibiting caspase3. It is highly expressed during fetal development and in most tumors (Cheung et al., 2013). In our study, Birc5 is upregulated in the neural tissue of DN. We hypothesized that Birc5 may play an important role in the occurrence and development of DN through cell cycle and apoptosis pathways.

Bub1 encodes a serine/threonine–protein kinase that plays a central role in mitosis. BUB1 involved in several pathways and played different roles in them (Breit et al., 2015). In the cell-cycle pathway, Bub1, MPS1, Bub3, Mad2, and Cdc20 formed a mitotic checkpoint complex (MCC), which leads to the inhibition of APC/C (Shi et al., 2011). In progesterone-mediated oocyte maturation pathway, Bub1 is phosphorylated by target Rsk downstream of MAPK and inhibits the function of the checkpoint effector Cdc20/fizzy with the complex Mad1/2/3 (Zhang et al., 2017).

Cdk1, cyclin-dependent kinase 1. The protein encoded by this gene belongs to the Ser/Thr protein kinase family. It plays a key role in controlling the eukaryotic cell cycle by regulating the centrosome cycle and the onset of mitosis (Santamaría et al., 2007; Jones et al., 2018). By linking with multiple interphase cyclins, Cdk1 promotes the G2-M transition and regulates the G1 process and G1-S transition (Prevo et al., 2018). During cell proliferation, G2-M phase CDK1-mediated FOXO1 phosphorylation inhibits the interaction between FOXO1 and 14-3-3 protein, thereby promoting FOXO1 nuclear accumulation and transcription factor activity, resulting in neuronal cell death after mitosis (Yuan et al., 2008). At the same time, phosphorylation of CALD1 affects the migration of Schwann cells during peripheral nerve regeneration.

Both Ccnb1 (cyclin B1) and Ccnb2 (cyclin B2) are members of the cyclin family, precisely the b-type cyclin. It is essential to control the cell cycle during the G2/M (mitotic) transition (Huang et al., 2013). Currently, few studies have been conducted on CCNB1, CCNB2, and their roles in DN. Zhang et al. reported that db/db mice showed upregulation of CCNB2 at mRNA levels, suggesting that it may cause diabetic nephropathy by interfering with G2/M phages (Zhang et al., 2017).

In addition, our data revealed the details of the infiltration of the 22 immune cells in DN for the first time. The proportion of T cells CD4 memory resting and dendritic cells resting in the DN group was higher, while the proportion of macrophage M2 was lower. Studies have found that CD4 + memory T cells are associated with the onset of type 1 diabetes. Quantification of CD4 + memory T cells can be used as an immunomarker for the diagnosis of type 1 diabetes (Orban et al., 2014). Gao et al. indicated that in high blood sugar state, the number of dendritic cells in the cornea of mice decreased, resulting in a decrease of CNTF, which affected the regeneration of sensory nerves (Leppin et al., 2014). Studies have confirmed that direct contact between dendritic cells and basal nerve plexus triggered nerve fiber damage, resulting in diabetic polyneuropathy (Gao et al., 2016). Macrophage M2, also known as alternatively activated macrophage, can downregulate the immune response by secreting the inhibitory cytokine interleukin-10 (IL-10) or tumor growth factor-beta (TGF-β), etc., clearing apoptotic cells and reducing inflammation (Roszer, 2015). Experiments have confirmed that the macrophage phenotype of diabetic peripheral neuropathy sufferers was characterized by reduced production of pro-inflammatory chemokines MCP-1 and IL-10, and the lack of IL-10 and TGF-β will increase the risk of diabetic peripheral neuropathy (Alvarado-Vázquez et al., 2019). At the same time, we further confirmed the relationship between key genes and immune infiltration. The results showed that the relationship between the five hub genes and immune infiltration was consistent, and they had a strong relationship with T cells CD8, T cells CD4 naive, T cells CD4 memory resting, monocytes, and dendritic cells resting. The results demonstrated that key genes and immune infiltration may play important roles in the progression of DN.

In conclusion, we screened 318 DEGs related to DN based on the GEO database and then screened out five hub genes through a variety of bioinformatics methods. The expression levels of five hub genes were confirmed by qRT-PCR. In addition, the correlation analysis of 22 immune cell infiltrations in DN samples revealed that five hub genes may affect the progress of DN through various biological functions and pathways. This research helps to further understand the development mechanism of DN and provide new ideas for the discovery of DN drug targets. In the future, we need larger genetic data and experimental research to confirm the findings of this study.

## Data Availability

The datasets presented in this study can be found in online repositories. The names of the repository/repositories and accession number(s) can be found in the article/Supplementary Material.
